# Lactic Acid Fermentation of Cereals and Pseudocereals: Ancient Nutritional Biotechnologies with Modern Applications

**DOI:** 10.3390/nu12041118

**Published:** 2020-04-17

**Authors:** Penka Petrova, Kaloyan Petrov

**Affiliations:** 1 Institute of Microbiology, Bulgarian Academy of Sciences, Acad. G. Bonchev, Str. Bl. 26, 1113 Sofia, Bulgaria; 2 Institute of Chemical Engineering, Bulgarian Academy of Sciences, Acad. G. Bonchev, Str. Bl. 103, 1113 Sofia, Bulgaria

**Keywords:** cereals, dietary fibres, fermentation, lactic acid bacteria, pasta, pseudocereals, sourdough

## Abstract

Grains are a substantial source of macronutrients and energy for humans. Lactic acid (LA) fermentation is the oldest and most popular way to improve the functionality, nutritional value, taste, appearance and safety of cereal foods and reduce the energy required for cooking. This literature review discusses lactic acid fermentation of the most commonly used cereals and pseudocereals by examination of the microbiological and biochemical fundamentals of the process. The study provides a critical overview of the indispensable participation of lactic acid bacteria (LAB) in the production of many traditional, ethnic, ancient and modern fermented cereals and beverages, as the analysed literature covers 40 years. The results reveal that the functional aspects of LAB fermented foods are due to significant molecular changes in macronutrients during LA fermentation. Through the action of a vast microbial enzymatic pool, LAB form a broad spectrum of volatile compounds, bioactive peptides and oligosaccharides with prebiotic potential. Modern applications of this ancient bioprocess include the industrial production of probiotic sourdough, fortified pasta, cereal beverages and “boutique” pseudocereal bread. These goods are very promising in broadening the daily menu of consumers with special nutritional needs.

## 1. Introduction

The word “cereal” originates from the Latin name of the ancient Roman goddess of harvest and agriculture, Ceres. In reality, cereals were domesticated nine thousand years before the founding of the Roman Empire. The first agricultural revolution began with the breeding of cereal grasses, the ancestors of emmer, einkorn and barley [1]. The first piece of evidence for cereal cultivation was einkorn grains found in southern Turkey and Syria, dating approximately 10,600–9900 BC [2]. At the same time, Chinese farmers have planted the predecessors of modern millet and rice and used human-made floods and fires to enhance the plant growth [3]. Sorghum was first cultivated in the region of Ethiopia over 5000 years ago [4]. Einkorn is still grown as a relict in some countries of South and Southeastern Europe, Asia, North Africa, Ethiopia and Italy (farro).

Today, according to the statistics of the UN Food and Agriculture Organization [5], the global consumption of cereals will increase from 2.6 bln tonnes in the base period (2017) to 2.9 bln tonnes in 2027, driven mainly by higher feed use (+167 Mt) followed by food use (+151 Mt). The largest among pseudocereals is the harvesting of buckwheat (1.5 Mt produced in Russia, and 0.3 Mt in European Union countries), followed by quinoa production in Peru and Bolivia, reaching 67 and 79 tonnes in 2017. In the next decade, the developing countries of Africa will expand the use of maize. In contrast, for developing countries in Asia, Latin America and the Caribbean, rice will remain the leading staple food as Asian countries.

Along with the development of ancient technologies for material goods production, spontaneously occurring biotechnologies contrived raw cereals food processing. Microbial fermentation, and especially lactic acid (LA) fermentation, became the most popular way to improve cereal taste, appearance and safety [6,7]. In addition, it improved the organoleptic characteristics, increased the nutritional value of starch-containing raw materials [8,9] and served as the primary method of preservation of food and as a strategy for reducing pathogenic bacterial contamination. LA fermentation contributes to the enrichment of human diet by an improvement of cooking with new flavours, aromas and textures. It allows the conservation of vast amounts of cereal food; enriches cereal substrates with protein, essential amino and fatty acids; plays a vital role in aflatoxin removal [10], and detoxification during pseudocereal processing [11]. LA fermentation of starch-containing raw materials leads to the production of a larger quantity of vitamins (group B and K), the amino acid lysine, folate and micronutrients in the fermented products [12,13]. Due to their unique beneficial properties, amylolytic lactic acid bacteria (LAB) can participate in the fermentation of probiotic starch-containing and hypoallergenic infant foods [14,15]. Recently, their role in type 2 diabetes prevention was proved [16].

Even though until recently LAB fermentation capacity was mainly associated with dairy products, many data proved that LABs have a crucial role in the preparation of dozens of traditional cereal-based foods worldwide [17,18]. Recent studies have found that LAB isolated from cereal foods have genetic prerequisites for the synthesis of enzymes involved in starch and prebiotics hydrolysis [19,20,21,22]. The present review of LA fermentation of cereals and pseudocereals has the following aims: to observe LA fermented foods worldwide focusing on their LAB content diversity and to reveal the genetic and biochemical peculiarities of the participating strains with beneficial effects on human health. A schematic overview of some traditional and modern technologies for fermented cereal products manufacturing was included.

## 2. Methods

The authors have searched the databases of PUBMED, SCOPUS, Google Scholar, CAZy, SpringerLink, Taylor and Francis, Wiley Online, Science Direct, EMBASE and CINAHL, by the use of the Boolean term “and” with the combination of the following search terms: cereals, pseudocereals, lactic acid fermentation, lactic acid bacteria, LAB fermented foods, macronutrient, glycoside hydrolase, starch, probiotic and fibres. The criteria for inclusion of the articles were as follows: their relevance, the double-review and the English language of publication. The exclusion criteria were: no double-reviewed, issued in a language other than English and published before 1980. The total search included 386 scientific publications, books and book chapters, 206 of which were excluded. One hundred seventy-nine works, published between 1980 and 2020, and one official web-site were extracted as fulfilling the including criteria.

## 3. Cereals and Pseudocereals Used for Food Production by LAB Fermentation

### 3.1. Cereals—Modern and Ancient Plant Types

**Figure 1:**
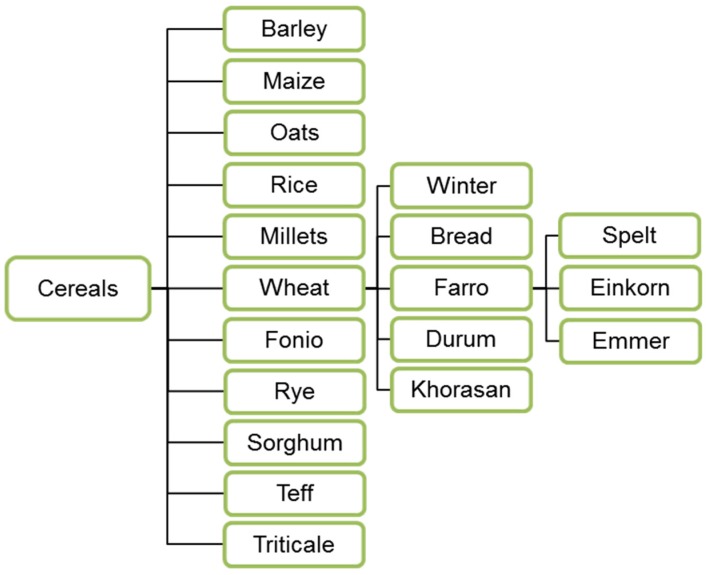
The most frequently used cereals for feed and food processing.

The nutritional values of traditional cereals such as wheat, maize and rice are due to the content of carbohydrates and proteins (Table 1), zinc, iron and B-vitamins [23,24,25,26,27]. Several species are most commonly used and distributed both as food and as a substrate for lactic acid fermented products (Figure 1).

In addition, an important feature of some cereals, such as barley, oats, rye and millet, is the high content of arabinoxylans and β-glucans, known as prebiotic fibre [28,29,30,31]. This is valid also for ancient grains like sorghum, einkorn and spelt [32]. Emmer has nutritional properties that are lacking in other cereals: 3 to 4 times higher β-carotene; twice the amount of vitamin A (retinol); thrice to four-fold lutein; four- to five-fold riboflavin [33]. Due to its low glycaemic index, and high satiety value is particularly suitable for special diets in cases of allergy, intolerance, sensitivity and diabetes [34].

Today innovative varieties of cereals are being created that have unique qualities, such as Khorasan (trademark Kamut^®^), which can accumulate three times higher amount of selenium than ordinary wheat [35]. The coloured (blue or purple) wheat is rich in anthocyanins (13.9 mg/100 g) that release free radicals and inhibit the oxidation of human lipoprotein cholesterol and possess anti-ageing effects [36].

*Eragrostis teff* is high in iron, which correlates with a higher level of haemoglobin and the absence of anaemia among teff consumers, including pregnant women [36,37,38]. Osteoporosis prevention is due to the teff’s extremely high content of magnesium/kg, or five to six times higher than in wheat [39]. Teff refers to gluten-free cereals and entirely lacks T-cell–stimulatory peptides [40]. Since the nutritional value of teff is similar to that of wheat, it is an appropriate substitute for gluten-containing cereals for patients with celiac disease [41].

However, the oldest African cereals are acha, also known as white fonio (*Digitaria exiliis*), and iburu, also known as black fonio (*D. iburua*). LA fermentation of white fonio types causes slight swelling, as starch partially leaches from the fonio starch granules [42].

### 3.2. Pseudocereals

**Figure 2:**
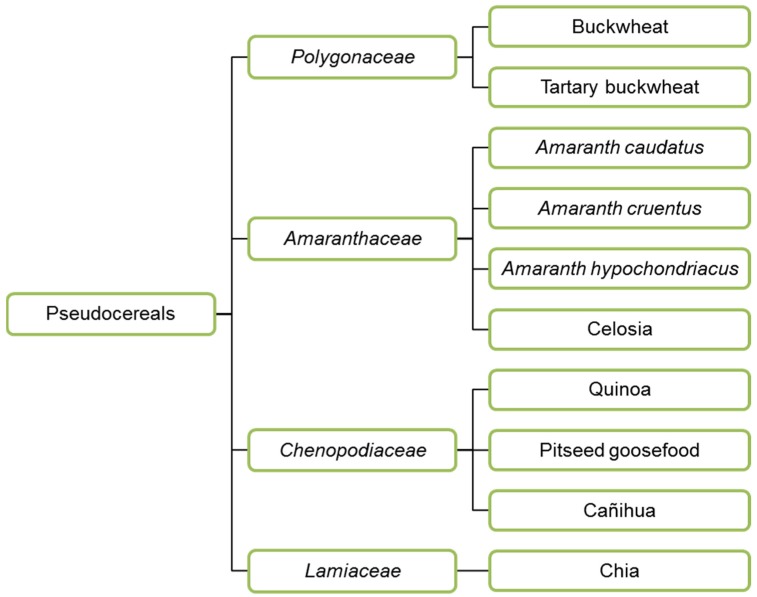
Taxonomy of pseudocereals.

In addition to cereals, pseudocereals are also used to make feed and food. Buckwheat (*Polygonaceae*), amaranth, quinoa (both *Amaranthaceae*) and chia (*Lamiaceae*) (Figure 2) are the most widely used pseudocereals. Amaranth and quinoa were essential food crops in Latin America, cultivated by Aztecs, Maya and Inca in the past. Buckwheat was cultivated around 6000 BC in Asia, first in Yunnan and Tibet, grown on arid soils on which very few plants can thrive [43]. Then it was spread to India, Japan, Europe and North America. Today, buckwheat is a viral seed in Europe, and widely consumed in the form of porridge in Russia, Ukraine, Belarus, Estonia and pancakes in France and Belgium (Wallonia). Buckwheat seeds are a source of several health-promoting substances: high vitamin content, polyphenols that may prevent cardio-vascular diseases and cancer, and fagopyritols, positively affecting non-insulin dependent diabetes [44].

Amaranth (*A. hypochondriacus* and *A. cruentus*) is an annual herbaceous plant used for grain. It belongs to the pseudocereal group widely distributed in all latitudes falling into four plant groups: grain, vegetable, decorative and weed. The grains contain high-quality protein rich in lysine and sulphur-containing amino acids [45]. Raw seeds are rich in harmful saponins and phenolic ingredients, but thermal treatment substantially reduces them [25].

Quinoa (*Chenopodium quinoa*) originates from the Andes. Seeds contain 14–18% protein with well-balanced amino acid composition, especially lysine, a good source of Group B vitamins, essential fatty acids, antioxidants and trace elements [25].

Chia (*Salvia hispanica*) belongs to family *Lamiaceae* and is spread in South American countries like Mexico and Guatemala. The seeds of chia contain 25–30% extractable oil, rich in ω-3 fatty acids (55%), ω-6 (18%) and ω-9 (6%). Ω-3 fatty acid is known to decrease the risk of major cardiovascular events such as arrhythmias, myocardial infarction, sudden cardiac death and coronary heart disease [46].

## 4. LAB Cereal Fermentation: Microbiology and Biochemistry of the Process

Lactic acid bacteria are a beneficial group of microorganisms that can preserve food by lactic acid fermentation. Probably the LAB was used in yoghurt-making, dating back to 4000 years BC when the Thracians domesticated sheep [47]. LAB are heterotrophic microorganisms and have high nutritional requirements for amino acids and vitamins because they have lost much of their biosynthetic potential in the process of evolution. For this reason, they only develop in nutrient-rich environments. They are usually found in milk and dairy products but can be isolated from plant or animal sources—leaves, fruits, roots, faeces and compost [19]. They widely participate in cereal fermented products [6,7,8,17,18,22], as well as in spontaneously fermented amaranth, quinoa and chia sourdoughs [48,49,50,51,52].

### 4.1. The Beneficial Impact of Cereal-Fermenting LAB on Human Health

Some LAB types, mainly lactobacilli, inhabit the gastrointestinal tract (GIT) of humans possessing probiotic effects. Lactobacilli and bifidobacteria maintain the balance between individual microbial species in GIT, which is the result of their production of bacteriocins, and other antimicrobials [53].

LAB make the food easily digestible, decreasing the level of high-chain carbohydrates and some indigestible poly- and oligosaccharides [19]. They also improve the availability of micronutrients iron, zinc and calcium [54]. Importantly, LAB adds functional characteristics to the cereal food composition. At first place, in course of the fermentation, LAB produce antimicrobial substances that inhibit pathogenic bacteria growth and prevent bacterial toxin formation [55]. Converting starch, they synthesize also essential short-chain fatty acids, and other low molecular organic components, like amino acids and B vitamins [19].

Many authors report an impact on improving human health status. LAB optimize weight gain, reduce serum glucose and total cholesterol levels [56]. LAB probiotics have been shown to have anti-inflammatory effect on intestine, because they are able to perform gluten detoxification in celiac patients [57]. During cell components hydrolysis, LAB liberate phenolic compounds and increase the antioxidant activity of the fermented food [58,59]. LAB alleviate cardiovascular and diabetes disorders by γ-aminobutyric acid (GABA), (ACE)-inhibitory peptides and menaquinones synthesis [60,61,62]. It was shown that the bioactive peptides obtained during LA fermentation of cereals, such as wheat, rye and barley and pseudo-cereal amaranth have anti-cancer activity [63].

### 4.2. LAB Taxonomy

Modern bacterial taxonomy classification refers to 6 LAB families and 39 genera, the valid LAB genera including *Lactobacillus*, *Leuconostoc*, *Pediococcus*, *Lactococcus* and *Streptococcus.* The genus *Bifidobacterium* is not present in the classification scheme because it is LAB associated, but belongs to phylum *Actinobacteria*. Although truly belonging to *Lactobacillales*, the genera *Aerococcus*, *Sporolactobacillus*, *Tetragenococcus*, *Vagococcus* and *Weissella* are considered as “more peripheral”. LAB morphology varies from long and thin rods (*Lactobacillus* and *Carnobacterium*) to cocci, often bound in a chain (*Aerococcus*, *Alloiococcus*, *Pediococcus*, *Oenococcus*, *Tetragenococcus*, *Vagococcus*, *Leuconostoc*, *Weissella*, *Streptococcus*, *Lactococcus*, *Globicatella* and *Lactosphaera*.

### 4.3. Starch Fermentation by Amylolytic Lactic Acid Bacteria (ALAB)

**Figure 3:**
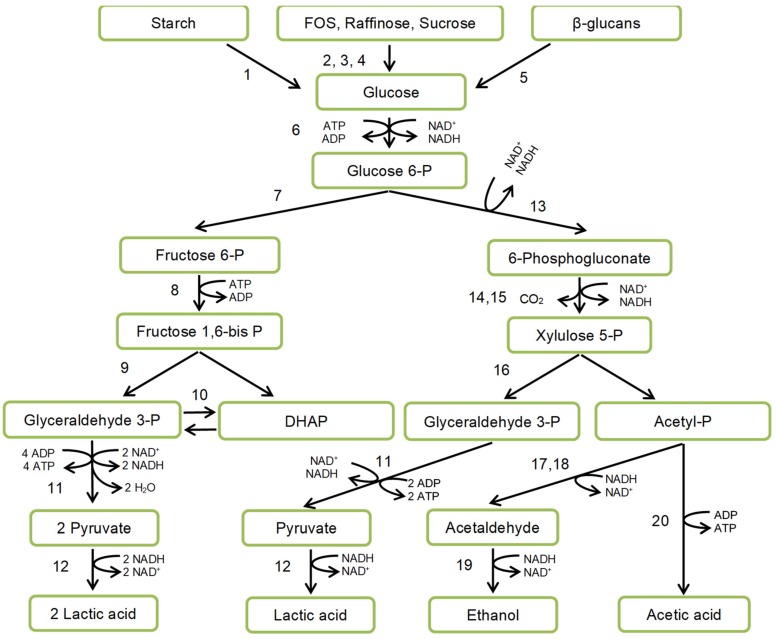
Scheme of lactic acid fermentation. Designations: DHAP—dihydroxyacetone phosphate; enzymes: 1—amylase; 2—fructosidase; 3—galactosidase; 4—sucrase (invertase); 5—β glucanase; 6—hexokinase; 7—phosphoglucose isomerase; 8—6-phosphofructokinase; 9—fructose-biphosphate aldolase; 10—triosephosphate isomerase; 11—glyceraldehyde 3-phosphate dehydrogenase, phosphoglycerate kinase, phosphoenolpyruvate hydratase, pyruvate kinase; 12—lactate dehydrogenase; 13—glucose 6-phosphate dehydrogenase; 14—6-phosphogluconate dehydrogenase; 15—ribulose 5-phosphate 3-epimerase; 16—phospho-ketolase; 17—phosphotransacetylase; 18—aldehyde dehydrogenase; 19—alcohol dehydrogenase; 20—acetate kinase.

LAB catabolism is accomplished by substrate phosphorylation. There are two main routes of substrate utilization: homo- and heterofermentative (Figure 3) differing in the presence or absence of key enzymes of the Embden-Meyerhof-Parnas pathway (fructose 1,6-bisphosphate-aldolase), or pentose-phosphate pathway (phosphoketolase).

Amylolytic LAB (ALAB) are very often isolated from cereals and have participated in the production of fermented foods for centuries [64]. *L. amylovorus* is one of the first described new LAB species able to degrade starch, along with other probiotic carbohydrates [65,66]. However, ALABs are relatively rare since, so far, representatives of only four LAB genera (*Lactobacillus*, *Lactococcus*, *Enterococcus* and *Streptococcus*) produce lactic acid directly from starch as a sole carbon source [23]. Considering the metabolic and genetic peculiarities of ALAB, several isolates represent new species: *L. amylophilus*, *L. amylovorus*, *L. amylolyticus*, *L. amylotrophicus* and *L. manihotivorans*. Most of these new species consist of one or two strains only; few of them have undergone additional reclassification. Different LABs known as amylolytic include *L. acidophilus*, *L. fermentum*, *L. plantarum*, *L. pentosus*, *L. paracasei*, *P. acidilactici*, *Lc. lactis*, *Str. bovis*, *Leuc. mesenteroides* and *W. confusa*. In 1983, the presence of intracellular amylase was proved in two strains of *L. acidophilus* [67]. Later, two other amylolytic strains of the same species were isolated, but the enzymatic activity of the latter was associated with the cell envelope. In addition to cell-associated, lactobacilli possess extracellular amylase activity [68]. Later, several other amylolytic representatives of *L. fermentum* were isolated [69]. Extracellular amylase activity was detected in strain Ogi E1, whereas a cell-associated α-amylase acted in the Mw2 strain [70]. Genes encoding amylase and neopullulanase have also been found in other strains of *L. fermentum* [19].

*L. plantarum* is the most common ALAB species in fermented foods, displaying high extracellular or cell-bound amylase activity [71]. There is one report about plasmid-encoded enzyme hydrolyzing starch, amylopectin, glycogen and pullulan [72], while the other enzymes are chromosomal [73,74]. Twenty-five new amylolytic representatives of *L. plantarum* and six strains of *L. paraplantarum* were isolated by Turpin et al. (2011) [19]. *L. amylolyticus* is another amylolytic LAB species isolated from beer malt [75]. After DNA/DNA hybridization experiments, which proved that the strain is as a new species, the type strain DSM 11664 was deposited.

*L. amylotrophicus* is closely related to *L. amylophilus*. Genomic and phenotypic studies proved that *L. amylotrophicus* is a new species with an LMG 11400 (NRRL B-4436) type strain [73]. *L. manihotivorans* is a new homofermentative ALAB species containing a plasmid-localized *amyA* gene [76]. *L. paracasei* produces extracellular amylopulanase [77]. The *AmyA* genes of *L. amylovorus*, *L. plantarum* A6 and *L. manihotivorans* are 98% identical and are entirely different from *amyA* in other lactobacilli. The enzymes contain two main functional domains: catalytic (amino acids 1–474) and starch-binding domain SBD (amino acids 475–953) [78]. The catalytic domain is a conserved region for all enzymes of the GH13 family. A distinctive feature of these enzymes is the Starch-Binding Domain (SBD) that comprises almost 500 amino acids. It contains tandem repeats of 91 amino acids: 4 repeats in *L. manihotivorans* and *L. plantarum* A6 and 5—in *L. amilovorus*. The role of SBD is adsorption to raw starch granules and attachment of the enzyme to the substrate, which increases the concentration of substrate in the active site of the enzyme. Therefore, amylase without SBD cannot degrade raw starch. Another essential property of extracellular amylase is the presence of a signal peptide that provides transport of the polypeptide chain out of the cell before enzyme maturation. It contains mostly hydrophobic amino acids (36 in *L. amilovorus* and *L. plantarum* A6) and a typical AQA excision site [77,78].

*Lc. lactis* is present in sorghum, maize, wheat sourdough and *posol* [79,80,81]. Despite the presence of starch in these sources, only some isolates of lactococci are amylolytic [82,83]. Two genes of *Lc. lactis* are responsible for starch hydrolysis: *amyY* and *amyL* encoding extracellular and cytoplasmic amylases in the amylolytic *Lc. lactis* subsp. *lactis* B84, with AmyL as the key enzyme [81].

Two different strains of *Str. bovis* strains JB1 and 148 were found to be amylolytic. The intracellular amylase of *Str. bovis* 148 (AmyB) hydrolyses soluble starch to a large amount of maltotriose and a small amount of maltose, whereas the extracellular enzyme of the same strain (AmyA) hydrolyses starch to maltose and glucose [84]. The amylolytic activity of *Str. bovis* 25124 and *Str. macedonicus* is significant, but information on their starch-degrading enzymes is not available [80].

The application of new ‘omics’ molecular technologies allows the analysis of the individual enzymes’ activities engaged in food fermentation. All ALAB strains possess extracellular or cell-bound amylase activity enabling them to produce LA from starch. However, a recent examination of the whole genomes of lactobacilli revealed the presence of only 48 of the known 133 families of glycoside hydrolases [85]. In addition, amylase-encoding genes are usually present (75% of ALAB contain *α-amy* gene), but many of them do not express mRNA, due to mutation damages in the promoter, in the amylase catalytic domain or the sequence encoding of the signal peptide [77]. This observation suggests that other glycoside hydrolase enzymes are also involved in starch degradation. The engagement of the following genes in the starch conversion was proved by a transcriptomic approach: *agl* (encoding α-glucosidase), *glgP* (glycogen phosphorylase), *malP* (maltose-phosphorylase), *dexC* (neopullulanase), *malL* (oligo-1,6-glucosidase), *glgB* (1-4-α-glucan branching enzyme and *treC* (trehalose-6-phosphate hydrolase). All investigated representatives of the genus *Lactobacillus* (except *L. sakei*) and *P. acidilactici* own and express all these genes, while strains of *E. faecium* and *E. durans* produce predominantly amylases [19,22]. The co-transcription of *glgP* and *glgB* genes indicates that glycogen synthesis and starch degradation are a parallel phenomenon, and proves the interconnection between biochemical paths. Pediococci possessed three genes responsible for starch hydrolysis: *amy*—encoding amylase, *dexC*—encoding neopullulanase and *malL* for oligo-1,6-glucosidase, which hydrolyses the 1,6-glycosidic linkages of short-chain substrates. Amylolytic representatives of genus *Enterococcus* are presented in the maize beverage *pozol* [80], in rye flour, wheat, in barley grains [22] and African beverages *bushera* and *kishra* [86].

*Leuc. mesenteroides* ssp. *mesenteroides* and *Leuc. mesenteroides* ssp. *dextranicum* capable of hydrolysing starch presented in *bushera*, *gapi* and *ben-saalga* [87]. Amylolytic *W. confusa* was isolated from several African fermented foods and beverages. The species is also widespread in French sourdoughs, and recently, the full genome sequence of *W. confusa* LBAE C39-2 revealed the presence of genes encoding neopullulanase (588 aa), amylopullulanase (560 aa) and oligo-1,6-glucosidase (560 aa) [88].

### 4.4. Fibres Fermentation by LAB: The Prebiotic Effect

Prebiotics are glycans, unified by their ability to stimulate the growth of probiotic bacteria in the gastrointestinal tract. To qualify a fibre as a prebiotic it is necessary to meet at least three requirements: (1) to resist the hydrolysis or absorption in the upper GIT parts (stomach or small intestine); (2) to be selectively digested by beneficial bacteria in the colon by inducing their growth and activity; (3) to induce a beneficial effect on the health of the consumer [89]. The positive effect of dietary fibres on the status of health is due to the synbiotic interaction with the probiotic species in the colon. In this process, prebiotics positively influence the metabolic activity of probiotic bacteria serving as a carbon source (bifidogenic effect). The short-chain fatty acids and the lactic acid, providing acidic pH in the lumen are the main products of LAB carbohydrate conversion at the lower part of GIT. Acetate, propionate and butyrate, produced by LAB, provide additional energy sources to the cells of intestinal mucosa, while lactate is capable of increasing the amount of resistant starch (RS) [90].

The American Cereals and Grains Association reported that there are two main groups of prebiotic carbohydrates (dietary fibres): water-insoluble (cellulose, chitin, hemicellulose, hexoses, pentoses, lignin, xanthan gum and resistant starch), and water-soluble: beta-glucan, arabinoxylan, galacto- and fructooligosaccharides [91,92].

Cereals contain several types of fibre that fulfil the requirements for prebiotics as β-glucan, arabinoxylan, galacto- and fructooligosaccharides. Raw oats, unrefined wheat and barley are the richest natural sources of prebiotic fibre among cereals. All cereals contain cellulose, hemicellulose and starch. Hexoses are abundant in wheat and barley, pentoses—in rye and oat; RS—in high amylose corn, barley and high amylose wheat. Rice is a plant with naturally low RS content ranging from 1.2% in white rice to 1.7% in brown rice [93]. However, high amylose rice varieties have been developed [94] that possess increased RS content up to 7.6% after cooking [95].

Resistant starches are prebiotic fibre, since the top of the GIT and small intestine do not absorb them due to high amylose content (60–70%), or modified structure [96]. They are known to alleviate metabolic disorders such as diabetes and hyperlipidemia [94], to participate in colon carcinogenesis prevention [97] and to stimulate LAB growth [98,99]. According to Birt (2013), there are five different types of RS [93]. The first class of resistant starch (RS I) is physically non-degradable starch contained in whole grains or coarsely milled seeds with high protein content. In this case, water does not successfully penetrate the starch granules enough to make them enzymatically hydrolysable. RS I-containing foods are whole-grain bread and durum wheat pasta, obtained by extrusion [100,101]. RS II is starch that contains B- or C-type polymorph in its natural granular form (in maize with high amylose content, uncooked potato, green banana and ginkgo). The high amylose starch has a higher temperature of gelatinization and after cooking at a lower heat this starch retains a crystalline structure that makes it resistant to enzymatic digestion. Type RS III is known as retrograded amylose and starch, obtained after processing of starch-containing foods (bread, pasta, rice). During the heat treatment and subsequent cooling, raw materials in these foods undergo degeneration—recrystallization of the amylose and amylopectin, which makes the starch resistant to enzymatic hydrolysis. RS IV are chemically modified hydrolysed starches with high crosslinking degree and low swelling ability [102]. RS V presents starch (both amylose and amylopectin) that is in a complex with fatty acids and fatty alcohols and forms helical structures that prevent starch binding and hydrolysis. RSV is thermally stable [103].

Xyloglucans possess different structures according to the number of (1→4) linked β-D-glucose residues in the branching. The prebiotic characteristics of xylooligosaccharides (XOS) include their ability to optimize colon and metabolic function and manage weight by reducing food intake [104]. Arabinogalactan is a polymer composed of arabinose and galactose residues in a furanose configuration linked with β-(1→5) and β-(1→6) glycosidic bonds. Its fermentation by members of the genus *Bifidobacterium* and *L. acidophilus* increases the amount of produced butyrate and propionate. Arabino-XOS (AXOS) show antioxidant activity in vitro and lead to elevated levels of butyrate, acetate and propionate in stools in vivo [105]. Fermentation of branched arabinose-XOS from wheat flour increases the selective growth of bifidobacteria [89].

Fructooligosaccharides (FOS) and inulin-type fructans are water-soluble prebiotic fibres widely spread in wheat and barley. It has been shown that after a two-week intake of inulin, bifidobacteria become dominant in faeces due to increased β-fructosidase activity. Thus, FOS and inulin prevent intestinal infections by inhibiting pathogens, regulating the intestinal immune system, increasing immune response; optimizing colon function and metabolism by producing short-chain fatty acids, and increasing the absorption of calcium [106,107]. Other glucose polymers with an apparent positive effect on consumers are β-glucans. It has been proved that the addition of β-glucans leads to increased growth of the probiotic strains *L. acidophilus* LA5, *L. plantarum* WCFS1, *L. plantarum* CETC 8328 and *L. fermentum* CECT 8448 in vitro [108].

Consumption of all fibres increases the number of *Bifidobacterium* and *Lactobacillus* cells. Acetate, propionate, butyrate and valerate are valuable metabolites produced during starch degradation by them, at the expense of less formed methanol and ethanol. SCFA are rapidly absorbed and metabolized by colonocytes, liver or other tissues [92]. Similar to the enzymes involved in starch utilization, the enzymes involved in other prebiotic fibre consumption belong to the glycoside hydrolase family (GH). Fructan-β-fructosidase (EC 3.2.1.80) and β-fructofuranosidase (EC 3.2.1.26) ensure inulin and FOS hydrolysis in *L. paracasei*, *L. casei*, *L. plantarum*, *L. pentosus*, *L. ruminis* and *L. acidophilus*. β-D-Xylosidase (EC 3.2.1.37), and α-L-arabinofuranosidase (EC 3.2.1.55) are responsible for XOS and AXOS utilization by *L. brevis* and *L. rossiae* [109].

## 5. LAB Fermented Cereal Foods and Beverages around the World

Lactic acid fermentation is a common, easy and cheap way to process starch-containing food. LAB fermentation enhances the nutritional and organoleptic value of food products. The first important improvement is in the sensory characteristics of foods. LA fermentation of cereal substrate obtains products with typical sour-sweet taste and delicious buttery aroma, such as bread, loaves, confectionery, pastes, noodles and gruels, or semi-digested beverages and complementary foods for infants and children. Several recent studies have reported the successful formulation of pseudocereal-containing gluten-free cereal-based products such as bread, pasta and confectionery products [110,111,112,113].

Starchy biomass is an alternative to glucose and a desirable cheap and renewable source in biotechnologies for LA production [77,114]. However, the leading worldwide application of ALAB is their involvement in the production of fermented foods and beverages, which are the staple food for the people in many regions of the world.

Table 2 observes the most frequently consumed fermented foods and beverages around the world made from conventional grains, with particular attention to ancient types of wheat and pseudocereals, and LAB participating during the fermentation process.

All types of fermented sourdoughs for bread making are vitally important fermented wheat flour products. In the first place, there is the sourdough of Europe, North America and Australia, where the microflora is dominated by heterofermentative, or the so-called “sourdough” lactobacilli. Among them, the most widespread include *L. sanfranciscensis*, *L. pontis*, *L. panis*, *L. paralimentarius* and many others [115,116,117,118,157]. Nowadays, sourdough microbiota accounts for about 50 different species, but it is very likely that a non-identifiable and perhaps new sourdough LAB exists [116]. There are dynamics in the sourdough LAB population regardless of the type of flour [158]. In all cases, the species belonging to the genera *Enterococcus*, *Lactococcus* and *Leuconostoc* dominate in the beginning, then we have the LAB species of *Lactobacillus*, *Pediococcus* and *Weissella* genera, followed by a predominance of heterofermentative species *L. sanfranciscensis*, *L. fermentum*, *L. pontis* and *L. plantarum* [117].

Wheat and barley flour are the raw material in addition to soybeans in the making of the Japanese *hamanatto* [121]. Moulds and LAB of genera *Streptococcus* and *Pediococcus* ferment the soy nuggets [18].

*Jalebies* are pretzel-like, syrup-filled confections prepared from deep-fried, fermented wheat-flour dough and consumed in India, Nepal and Pakistan [122]. In China, mantou is a traditional bread, prepared by steaming LAB and yeast-leavened wheat dough, often filled with sweets, meats and vegetables [140].

The cereal alternative to wheat is its minor and ancient varieties of einkorn and spelt. The starters *L. plantarum*, *L. sanfranciscensis* and *L. brevis* participate in sourdough and bread fermentation [124]. Coda et al. (2010) developed LAB starter cultures for farro (spelt) sourdough and manufacturing of a new functional beverage based on emmer flour [126]. It contains *L. brevis*, *W. confusa* and *L. plantarum*. Metabolomics studies also demonstrated a significant improvement of the functional and sensorial profile of Khorasan based fermented foods by the use of *L. plantarum* strains producing volatiles such as alcohols, carbonyls, dodecanoic acid, 1,3-hexadiene and polyphenolic compounds [127].

Einkorn is the source of *boza* preparation in Bulgaria [8]. In Germany, farmers primarily cultivate spelt for the production of traditional bread. The autochthonous microbiota of spelt is diverse and includes *L. brevis*, *W. confusa* and *L. plantarum* [31].

Fermented sorghum and millet serve to obtain alcoholic and non-alcoholic drinks in many African countries. They are the grain substrates for the traditional alcoholic drink *muramba* and the non-alcoholic beverage *bushera*. LAB engaged in *bushera* production belong to genera *Lactococcus*, *Leuconostoc*, *Lactobacillus*, *Weissella* and *Enterococcus* [87]. *Kunun-zaki* is another millet-fermented beverage widely consumed in Northern Nigeria, while Ethiopian consumers are quite familiar with barley foods and drinks such as *ingera*, *kita*, *dabo*, *kolo*, *genfo*, *beso*, *chuko*, *shamet*, *tihlo*, *kinche* and *shorba*. Barley, roasted-milled grains or flours are the substrate basis for LAB fermentation.

*Injera* is traditional fermented Ethiopian bread made from barley or teff flour, water and starter—a small portion from previously fermented dough [151]. The mixture is usually allowed to ferment for three days by *L. buchneri* and *P. pentosaceus*. Importantly, LAB strains isolated from teff *injera* display high phytase activity. The use of decreased phytate contents in cooked *injera* improves human zinc absorption [154]. Other barley fermented foods are also known as functional. For instance, *genfo* serves as a good substitute for breast milk, *beso* is used in the case of GIT disorders and gastritis, while *genfo* and *kinche* improve the healing of broken bones and fractures [159].

*Ragi ambli* (*ambali*) is an Indian meal, made after fermentation of finger millet. The meal contains flour, buttermilk, salt, onion and curry leaves. Due to the high dietary fibre content in the finger millet [160], its spontaneously LA fermented drinks were found to alleviate children’s diarrhoea [161]. The effect of consumption of finger millet whole grain and bran was tested using a mice model fed on a high-fat diet by Murtaza et al. [162]. The results revealed that fibres in bran prevented obesity, improved the lipid profile of mice, and increased LAB content in the gut.

In Asia, the traditional bread is made of fermented rice (Philippines). The people in Nepal and India produce sweet fermented rice bread called *selroti* that contains *Lactobacillus* ssp., *Leuconostoc*, pediococci, enterococci and yeast as starter cultures [146]. The daily menu of people in the Indian subcontinent includes large amounts of acid-leavened bread and pancakes. *Idli*, *dosa* and *dhokla* are popular in Southern India and Sri Lanka. *Idli* is a savoury rice cake originating from the Indian subcontinent, popular as a breakfast food in Southern India and among the Tamils in Sri Lanka. *Idli* is a result of lactic fermentation of rice flavoured with black gram dhal [141]. *Dosa* is very similar to *idli* batter since both are products of natural lactic acid fermentation for 20 h and resulting in a reduction of sugars, complete hydrolysis of the oligosaccharides (stachyose and raffinose), decreased phytate content and increased thiamine and riboflavin content. After the process of LA fermentation, the *dosa* is fried to a crunchy pancake and is ready for consummation. *Dhokla* is similar to *idli* with differences in the rice sort. The starter culture for these meals contains *Leuc. mesenteroides* associated with the ingredients [140,141]. Several types of bread are prepared primarily by acid fermentation of rice flour dough.

*Jeung-pyun* is a fermented rice cake produced by mixing rice flour, water, sugar, salt and a little amount of the traditional Korean rice beer *makgeolli*. The last is used as a starter that is rich in amino acids, sugars and vitamins, and supports the growth of LAB species of genera *Lactobacillus*, *Weissella* and *Pediococcus* [142]. The Korean *kichudok* and the Philippine *puto* are leavened, steamed rice cakes that are similar to the Indian *idli*. Year-old rice is used to make *puto*. *Kichudok* is consumed on special occasions, while *puto* is intended for breakfast and as a snack [140]. Indonesians prepare *brem*. It is a solid rice cake, with a sweet and slightly sour taste, containing over 65% glucose [139]. Thai rice-noodle *khanomjeen* is also prepared from acid-fermented rice, soaked in water for three days and then ground into a pulp. The *Lactobacillus* and *Streptococcus* genera are involved in the process [144].

LA fermentation of cereals preserves fish, combined with rice, millet flour and sugar syrup. Such cereal/fish fermented meals include the Korean *sikhae* and the Philippine *balao-balao*. The species responsible for LA production in *sikhae* are *Leuc. mesenteroides* and *L. plantarum* [137].

*Burong-isda*, prepared in the Philippines from fish and rice, contains *L. brevis* and *Streptococcus* spp.; pediococci ferment *pla-ra* (made of pork, garlic, salt and rice) in Thailand; *nham* contains *P. cerevisiae*, *L. plantarum* and *L. brevis* as starters; the Vietnamese *nem-chua* is a similar meal, made with the participation of *Pediococcus* spp. and *Lactobacillus* spp. [163].

Maize meals fermented by LAB are typical for Africa and South America. The sour maize-based *mahewu* (*amahewu*) are traditional gruel/beverages in South Africa. The West African *ogi* is a staple porridge prepared from fermented maize, sorghum or millet; *togwa* is fermented maize/sorghum gruel widely consumed in Tanzania; *munkoyo* is a traditional Zambian maize fermented gruel, prepared with the addition of *Rhynchosia heterophylla* root extract for its amylases [130]. Famous LAB fermented meal in Mexico is maize porridge/beverage *atole agrio* [128,129], while *pozol* is a refreshing LAB fermented drink [80].

## 6. Traditional Microbial Processes with Modern Applications: LAB Fermented Rye Bread, Pasta and Cereal Beverages

### 6.1. Rye Bread Production Technology

Rye is considered a minor cereal since its world production does not exceed 1% of the world grains, and its consummation is about 10–30 kg/year per capita, even more in countries with constant rye consumption like Russia, Poland, Denmark, Finland, Germany and the Baltic states. However, rye bread is made, sought after and loved in many countries for its unique flavour and taste, and prolonged shelf-life provided by LAB strains activity [164,165,166].

**Figure 4:**
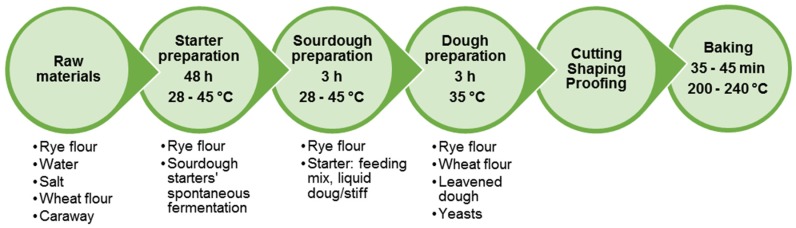
Flowchart of traditional rye bread production.

Figure 4 presents a flow chart of rye bread production by traditional technology using sourdough. It consists of the development of particular starter culture, sourdough and bread dough preparation.

Briefly, wholemeal rye, salt and water are mixed and allowed to ferment for 48 h to develop the growth of autochthonous LAB microflora of rye flour and to obtain the starter culture. The starter may contain *L. plantarum*, *L. delbrueckii*, *L. farciminis*, *L. casei*, *L. acidophilus*, *L. johnsonii*, *L. brevis*, *L. fermentum*, *L. buchneri*, *L. fructivorans*, *L. pontis*, *L. panis*, *P. pentosaceus*, *P. acidilactici* [115,118]. The process of starter development in Baltic countries includes an additional step of preliminary scalding of rye flour (heat treatment with hot water or steam), not performed during the processes used elsewhere [166]. After the preparation of a sweet (heated) scald, it cools down to 45 °C, a temperature, which is optimal for scald saccharification and the propagation of thermophilic LAB. Rye flour and water are mixed with the scald to obtain sourdough. The final dough contains rye flour, sourdough, wheat flour and glucose syrup. In many cases, yeasts are added to the fermented scald and sourdough mixture right before dough preparation. Dough fermentation lasts about 3 h, while the baking takes between 30 and 45 min.

### 6.2. Fermented Pasta Production Technology

**Figure 5:**
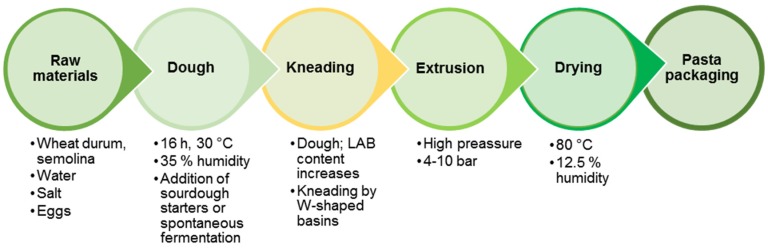
Flowchart of traditional pasta manufacturing.

The first data about the preparation of original Italian pasta date from the 13th century in Southern Italy—in Pisa (vermicelli) and Naples (macaroni). The pasta had become a staple food in the region of Naples by the 18th century, acquiring its present appearance as a dried product and becoming part of the cuisine of the entire Apennine Peninsula and many European countries [167]. Traditional pasta-making technology is based on the use of durum wheat (semolina), water, salt and eggs. It includes the stages of dough preparation, spontaneous fermentation, kneading, extruding and drying into sheets or various shapes (Figure 5).

A study of the dominant LAB participating in pasta spontaneous fermentation process showed that the participating strains belong to *P. pentosaceus*, *E. faecium*, *W. confusa*, *P. acidilactici*, *L. mesenteroides*, *L. citreum*, *L. fermentum* and *L. plantarum* [168]. An increase of the LAB population occurs in the process of kneading to extrusion in the traditional technological steps.

Today, available knowledge has introduced individual LAB-based biotechnological steps for fortified pasta production. After analysis of the LAB content of durum wheat, innovative applications of LAB improve the pasta-making process. Two steps have been added to pasta-making technology to achieve the manufacturing of pasta with decreased gluten content: fermentation of durum semolina by preparation of liquid culture of selected LAB species and freeze-drying of the starter [169]. A similar fermentation step of wheat semolina with *L. plantarum* results in riboflavin-enriched pasta [170]. The modified pasta was not different in its organoleptic properties from that obtained by the traditional process. Another advanced approach to obtaining gluten-free pasta is the substitution of durum semolina with pseudocereal flours of buckwheat, amaranth and quinoa flours as substitutes of wheat in pasta making [171]. Quinoa and corn flours were mixed in spaghetti manufacturing [172]. In both cases, the obtained product was with satisfactory quality, appearance and sensory properties.

### 6.3. Production Technology of LAB Fermented Cereal Beverages

In addition to bread and pasta where LAB contribute only to the pleasant odour, taste and improved quality of the product, the use of LAB fermented cereal beverages offers unique health-promotion. The beneficial prebiotic effect of eating cereals containing dietary fibres is complementary to the direct consumption of vital probiotic LAB.

*Boza* is a thick, sweet-sour, low-alcoholic beverage made in Eastern Europe and the Middle East, and popular in Bulgaria, Albania and Turkey. It has high LAB content with comprehensively established probiotic properties [8,120,173]. *Bushera* is a functional beverage, traditionally prepared in the Western Highlands of Uganda. It contains decreased phenolic and tannin content, and LAB of five genera, among them the probiotic candidate *L. brevis* [87,174]. *Kunun-zaki* is a sweet-sour and creamy drink, widely manufactured in Nigeria, similar to the gruels/drinks *mahewu* and *baganiya* [136].

Both adults and children consume the three beverages while LAB fermentation is in progress. They have similar preventive and therapeutic health effects. For instance, *boza* consummation assists in blood pressure balance, and also improves colonic health and decreases plasma cholesterol [8]. As to *bushera* and *kunun-zaki*, it is generally believed that they enhance lactation of nursing mothers and are drinks that may substitute mother milk due to their high nutritive and pro/prebiotic content.

**Figure 6:**
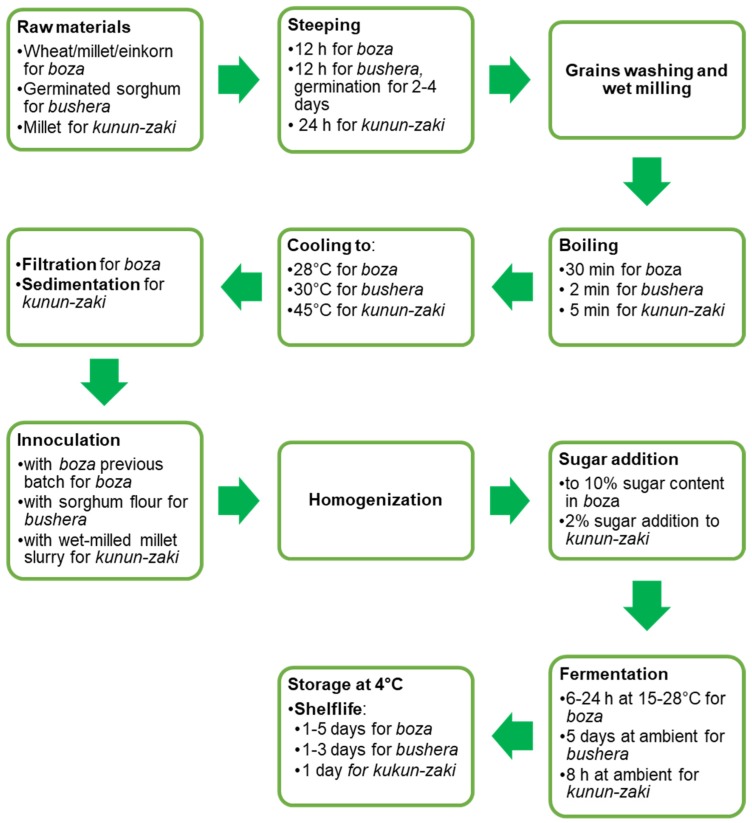
Generalized scheme of production of lactic acid bacteria (LAB) fermented cereal beverages *bosa*, *bushera* and *kunun-zaki*.

Notwithstanding their different starting raw material, *boza*, *bushera* and *kunun-zaki* have a similar flow-chart of production (Figure 6). That includes cereal soaking, germination, milling, boiling, sieving and LAB fermentation. The final beverages are also similar in appearance: *boza* is served straight, with cinnamon or cocoa, while *kunun-zaki* is usually flavoured with ginger.

## 7. LAB Pseudocereals Fermentation for the Development of Foods for Consumers with Health Problems

As mentioned above, pseudocereals are the modern substitute for gluten-containing cereals for people suffering from autoimmune enteropathy, or celiac disease. By their chemical content, pseudocereals possess lower starch but higher protein content compared to wheat. However, their seeds contain mainly proteins of albumin and globulin fractions (rich in essential amino acids) and do not contain the prolamins causing celiac disease. Dietary fibre content is similar to cereals. Pseudocereals are excellent in vitamin content, especially concerning riboflavin, thiamine, folate, pyridoxine and vitamin E [25].

The main application of pseudocereal seeds is in bread making. In many countries, buckwheat is used for gruels, porridges and pancake cooking. However, due to their poor palatability, many buckwheat products are made with sourdough, containing *P. pentosaceus*, *E. faecalis*, *W. cibaria* [175], *L. plantarum*, *L. salivarius*, *L. rhamnosus* [176,177] and *L. paracasei* [178]. Amaranth seeds participate in the manufacturing of iron-fortified bread [179]. In terms of bread qualities as volume and organoleptic properties, fermented buckwheat and quinoa were better than the control samples. In addition, LAB fermentation always improves the aroma quality of gluten-free products, for selected strains may generate specific volatile organic compounds in wheat sourdough.

Successful new biotechnology for antidiabetic food has appeared recently. The meal is produced in solid-state fermentation by the engagement of Tartar buckwheat flour and starter culture containing *L. paracasei* and *L. plantarum* [180].

Several studies have described the application of pseudocereals in gluten-free biscuit manufacturing using amaranth flour [110,111]. Progress in LAB fermented quinoa beverage and pasta was also recently reported [112]. By the use of a mouse model, it was found that quinoa pasta fermented by LAB prevents vitamins and minerals deficiency [113].

Original chia sourdough starter culture is developed by a selection of autochthonous LAB strains with affiliation to the species *L. plantarum*. Chemical analysis of chia sourdough showed high antioxidant effectiveness, and total phenolic compounds increased with 25% after 24 h of fermentation by *L. plantarum* [51].

## 8. Conclusions and Future Perspectives

When considering the beneficial effects of fermented cereals from LA on the human body, several factors need to be evaluated: (i) the beneficial content of the grains themselves, (ii) the probiotic properties of the LAB strains involved in the process, (iii) the biochemical changes that occur in the grain substrates during fermentation and (iv) the genetic and enzymatic capabilities of LAB strains that allow them to utilize grains’ carbohydrates. Thus, the individual health benefits of cereals and probiotic bacterial strains are combined into a unique synergistic effect.

This study elucidates the complex mechanisms of grain/LAB interaction, with special emphasis on the macro- and micronutrient content of grains, and the unique biochemical properties of amylolytic LAB strains participating in the food processing. Cereals and pseudocereals supply not only the basic nutrition of the human population but also provide valuable compounds such as fibre, antioxidants, vitamins, minerals and essential amino acids. In addition, LA fermentation produces a number of positive changes in the cereal food composition: enrichment of protein, amino acids and fatty acids content, toxin removal and partial hydrolysis of starch and gluten.

The relationship between LAB and cereals is the fine link between the bacterial strain and its preferable substrate. The investigation of the genetic and biochemical basis of these interactions revealed that the processing of cereals is mainly due to LAB genera that possess enzymatic systems for digesting various types of carbohydrates: starch and/or prebiotic fibres such as resistant starch, fructans, beta-glucans and xyloglucans. Moreover, the ability to consume prebiotic carbohydrates is one of the criteria for defining a strain as probiotic. Isolation and selection of such strains enable the development of new functional foods in which the beneficial properties of the probiotic strain are combined with the nutritional and beneficial value of the grains.

The consumption of ancient and endemic cereals and pseudocereal grain varieties leads to the improved status of various organs and systems in the human body and contributes to the prevention of cardiovascular, cancerous, metabolic and allergic disorders. That is why, currently, more and more research effort is focused on finding ancient and forgotten cereal crops. People are particularly drawn by the boutique bread made of einkorn, emmer or spelt, as well as the fermented beverages containing probiotic strains, probiotically fortified pasta and new ‘LAB processed’ pseudocereal goods. However, the LA fermentation features of all these crop variants have not been studied so far and need more serious investigation in the future.

LA fermented cereal goods provide the consumer with functional food products with a specific composition and physiological functions of health relevance. The development of various LA fermented cereal products with specific content may be of help to 795 million people with eating disorders worldwide, including 41 million overweight children. For most people with food allergies, especially for those with celiac disease, LA fermentation of cereals allows expanding the diversity of foods they can consume. Based on traditional ancient recipes, new LA fermented cereal alternatives with partially digested gluten or gluten-free products could be developed. Unambiguously, the purposeful development of new wholesome foods with nutritional, curative and prophylactic action will rely on the joint efforts of plant selection, nutrition science, medicine and biotechnology.

## Figures and Tables

**Table 1:** Cereals and pseudocereals grain/seeds content (g per 100 g).

Grain/Seed	Carbohydrates	Protein	Fat	Fibres
Wheat (bread)	71.2	12.6	1.5	12.2
Einkorn	65.5	15.8	4.2	8.7
Emmer	65.0	14.0	1.8	2.7
Spelt	53.9	14.6	2.4	10.7
Khorasan	52.4	14.7	2.2	9.1
Maize ^†^	74.0	9.4	4.7	7.3
Rice (white) ^†^	80.0	7.1	0.7	1.3
Barley	77.7	9.9	1.2	15.6
Proso Millet ^†^	72.8	11.0	4.2	8.5
Sorghum Millet ^†^	75.0	11.3	3.3	6.3
Oat ^†^	66.3	16.9	6.9	11.6
Rye	60.7	8.8	1.7	13.2
Teff (cooked)	19.9	3.9	0.7	2.8
Buckwheat ^†^	58.9	12.5	2.1	29.5
Amaranth ^†^	61.4	16.5	5.7	20.6
Quinoa ^†^	64.2	14.5	5.2	14.2
Chia ^†^	42.1	16.5	30.7	34.4

^†^ Millets, maize, rice and pseudocereals are gluten-free grains/seeds; oat contains less than 20 μg/g of gluten [27]. All other grains contain gluten in different amounts. Grains contain also essential vitamins of group B (riboflavin, thiamin, pyridoxine and niacin), as well as vitamin E, folate and pantothenate and microelements iron, zinc, magnesium, phosphorus and selenium [24].

**Table 2:** The most popular foods and beverages around the world obtained by lactic acid fermentation of cereals and pseudocereals.

Cereal	Product	Use	LAB Species	Country of Main Use	Reference
**Wheat and varieties**					
**Wheat**	Sourdough	Bread	*L. brevis*, *L. fermentum*, *L. buchneri*, *L. reuteri*, *L. frumenti*, *L. pontis*, *L. fructivorans*, *L. sanfranciscensis*, *L. panis*, *W. confusa*, *W. cibaria*, *L. casei*, *L. alimentarius*, *L. plantarum*, *P. pentosaceus*, *L. amylovorus*, *L. paralimentarius*, *L. acidophilus*, *L. acidophilus*, *L. delbrueckii*, *L. farciminis*, *L. mindensis*, *L. johnsonii*	Italy, Belgium, France, Portugal, Germany, Central Europe countries	[115,116,117,118]
	Boza	Thick beverage	*L. coryniformis*, *L. fermentum L. plantarum*, *L. pentosus*, *L. paracasei*, *Leuc. paramesenteroides*, *Leuc. sanfrancisco*, *Leuc. mesenteroides*	Bulgaria, Albania, Turkey	[8,119,120]
	Hamanato	Snack	*Streptococcus*, *Pediococcus*	Japan	[121]
	Jalebies	Pretzel	*L. plantarum*, *E. faecium*, *L. fermentum*, *L. paracasei*	India	[122]
	Kishk	Meal	*L. plantarum*, *L. brevis*, *L. casei*	Egypt, Syria	[123]
**Einkorn**	Sourdough	Bread	*L. plantarum*, *L. sanfranciscensis*, *L. brevis*	Italy	[124]
**Emmer**	Functional beverage	Drink	*L. plantarum*, *W. confusa*	Italy	[125]
**Spelt**	Bread	Bread	*L. brevis*, *W. confusa*, *L. plantarum*	Italy	[126]
**Khorasan**	Sourdough	Bread	*L. plantarum*	Italy	[127]
**Farro**	Acha, Iburu	Porridge Couscous	*P. pentosaceus*, *L. curvatus*, *L. plantarum*	Nigeria	[42]
**Maize**	Atole agrio	Porridge	*Weissella*, *Pediococcus*, *Lactococcus*, *Lactobacillus*	Mexico	[128,129]
	Broa	Bread	*Leuconostoc*, *L. brevis*, *L. curvatus*, *Lc. lactis* ssp. *lactis*, *E. durans*, *Ent. casseliflavus*, *E. faecium*, *S. equinus*, *Str. constellantus*	Portugal	[79]
	Busaa	Alcoholic drink	*L. helveticus*, *L. salivarius*, *L. casei*, *L. brevis*, *L. plantarum*, *L. buchneri*	Nigeria, Ghana	[17]
	Kenkey	Mush	*L. fermentum*, *L. reuteri*	Ghana	[17,87]
	Koko	Porridge	*L. plantarum*, *L. brevis*	Ghana	[17,87]
	Kwete	Beverage	*L. rhamnosus*, *Str. thermophilus*	Uganda	[15]
	Magu	Porridge	*L. delbrueckii*	RSA	[130]
	Mahewu	Beverage	*L. plantarum*, *L. fermentum*	RSA	[131]
	Pito	Drink	*Lactobacillus*	Nigeria, Ghana	[132]
	Pozol	Beverage	*E. faecium*, *Str. bovis*, *L. fermentum*	Mexico	[80]
	Togwa	Gruel	*L. plantarum*, *L. brevis*, *L. fermentum*, *W. confusa*, *P. pentosaceus*	Tanzania	[133]
**Millet**	Ambali	Porridge	*Leuc. mesenteroides*, *L. fermentum*, *Str. faecalis*	India	[134]
	Bagni	Alcoholic drink	*Lactobacillus* spp.	Russia	[135]
	Kunun-zaki	Thick beverage	*Lactobacillus* spp., *L. fermentum*	Nigeria	[136]
	Merrisa	Drink	*Lactobacillus* spp.	Sudan	[135]
	Sikhae	Fish meal	*Leuc. mesenteroides*, *L. plantarum*	Korea	[137]
**Sorghum**	Bantu	Beer	*L. delbrueckii*	RSA	[87]
	Kishra	Bread	*E. faecium*	Sudan	[86]
	Nasha	Porridge	*Lactobacillus*, *Streptococcus*	Sudan	[16]
	Ogi	Paste	*L. plantarum*	Nigeria	[70]
	Uji	Porridge	*Leuc. mesenteroides*, *L. plantarum*	Kenya	[17]
**Rice**	Adai/Vada	Snack	*Leuconostoc*, *LA cocci*	India	[138]
	Brem	Cake	*P. pentosaceus*, *E. faecium*, *L. curvatus*, *W. confusa*, *W. paramesenteroides*	Indonesia	[139]
	Dhokla	Cake	*Leuc. mesenteroides*, *Ent. faecalis*	India	[140]
	Idli	Cake	*Leuc. mesenteroides*, *Ent. faecalis*	India	[141]
	Jeung-pyun	Cake	*Leuc. lactis*, *Leuc. citreum*, *L. brevis.L. crustorum*, *L. fermentum*, *L. harbinensis*	Korea	[142]
	Khanom-jeen	Noodle	*Lactobacillus*, *Streptococcus*	Thailand	[143,144]
	Puto	Paste	*Leuc. mesenteroides*	Philippines	[140]
	Plaa-som	Fish/Rice	*Lc. garvieae*, *S. bovis*, *W. cibaria*, *P. pentosaceus*, *L. plantarum*, *L. fermentum*	Thailand	[145]
	Selroti	Bread	*Leuc. mesenteroides*, *E. faecium*, *P. pentosaceus*, *L. curvatus*	Nepal	[146]
	Tapuy	Beverage	*Leuconostoc*, *Lactobacillus*	Philippines	[17]
**Rye**	Sourdough	Bread	*P. pentosaceus*, *Streptococcus*, *Lactobacillus*, *W. paramesenteroides*	Germany, Serbia	[147]
	Sourdough	Bread	*Lc. lactis*, *L. paralimentarius*, *L. kimchii*, *L. sanfranciscensis*	Bulgaria	[81,148]
	Sourdough	Bread	*L. plantarum*, *L. brevis*, *L. plantarum*	Finland, Denmark, Norway, Sweden	[149,150]
**Barley**	Injera	Bread	*L. buchneri*, *P. pentosaceus*	Ethiopia	[151]
	Keribo	Beverage	*Lactobacillus* spp.	Ethiopia	[152]
	Miso/Soy	Paste	*E. faecium*, *E. durans*, *E. faecalis*, *P. acidilactici*, *P. pentosaceus*, *L. plantarum*, *W. confusa*	Japan, China	[153]
**Oat**	Probiotic drink	Beverage	*L. plantarum*	Bulgaria	[29]
**Teff**	Injera	Soft pancake	*L. buchneri*, *P. pentosaceus*, *Lb. pontis*, *Lb. plantarum*, *Leuc. mesenteroides*	Ethiopia	[154,155]
	Tella	Alcoholic drink	*L. pastorianumi*, *Leuc. mesenteroides*	Ethiopia	[135]
**Buckwheat**	Sourdough	Bread	*L. acidophilus*, *L. casei*, *L. plantarum*, *L. rhamnosus*, *L. salivarius*, *L. delbrueckii subsp. bulgaricus*	Poland	[156]
**Amaranth**	Sourdough	Bread	*L. plantarum*, *L. rhamnosus*, *E. mundtii*, *E. hermanniensis*, *E. durans*, *Leuc. mesenteroides*	Argentina	[48,49]
**Quinoa**	Sourdough	Bread	*E. hermanniensis*, *E. casseliflavus, E. mundtii*, *Lc. lactis*, *Leuc. citreum*, *L. plantarum*, *L. brevis*, *Leuc. mesenteroides*	Argentina	[50]
**Chia**	Sourdough	Bread	*L. plantarum*, *E. faecium*, *E. mundtii*, *W. cibaria*, *L. rhamnosus*, *Lc. lactis*	Argentina	[51,52]

## Abbreviations

ALAB	Amylolytic Lactic acid bacteria
GIT	Gastro-Intestinal Tract
GOS	Galactooligosaccharides
FAO	Food Agricultural Organization of the United Nations
FOS	Fructooligosaccharides
LA	Lactic acid
LAB	Lactic acid bacteria
p.a.	per annum
SBD	Starch-Binding Domain
SCFA	Short Chain Fatty Acids
